# Supplementary material for: Lysine methyltransferase SETD6 modifies histones on a glycine-lysine motif
Source: Epigenetics. 2019 Aug 1;15(1-2):26–31. doi: 10.1080/15592294.2019.1649529 (PMC6961689; doi:10.1080/15592294.2019.1649529)
Supplement: Supplemental Material [file kepi-15-1-2-1649529-s001.pdf]

Table S1: List of putative SETD6 substrates with predicted modification sites and known methylation sites identified by mass spectrometry (MS).

Substrate	GK^{me}DS		MS database GKDS (or GKDS-like) sites
ABCC6	808	Multi-drug resistance	
ADAMTS7	1200	Metallopeptidase	
ADGRL3	611	Cell adhesion	
ADGRL3	682	G-protein coupled receptor	
AHNAK2	38		AK^{994}DS; AK^{1819}DS; AK^{2974}DS; AK^{3139}DS; AK^{3304}DS; AK^{3634}DS; AK^{3799}DS; AK^{4129}DS; AK^{4459}DS
AHR	438	Nuclear receptor	
ALS2	677	Guanine exchange	
APAF1	100	Apoptotic peptidase	
C5orf42	1375	Joubert syndrome	
CELF4	154	RNA recognition	
COL12A1	2846	Collagen	
CSMD1	320		
DHX30	388	RNA helicase	
DMXL1	849	WD repeat	
DSPP	271	Dentin	
ERICH3	530	E-rich protein	GK^{203}KA
FHAD1	1077	Forkhead domain	
FNDC3A	970	Fibronectin	
GAPVD1	796		
GREB1	278	Estrogen signalling	
IL16	235	Interleukin	
KAT6B	393	MORF/MOZ2/MYST4 acetyltransferase	
KDM5A	1504	RBP2 lysine demethylase	
KIDINS220	1313	ATPase	
KIF26A	469	Kinesin	
KIF4A	995	Kinesin	
LRBA	1070	Anchor protein	
MAGEC1	74; 106	Tumour antigen	
MDN1	781	ATPase	GK^{781}DS
MLH3	982	DNA mismatch repair	
MROH7	714		
MUC5AC	937	Mucin	
OAS3	985	Protein synthesis	
PCDH18	988	Protocadherin	
PCDH8	915	Protocadherin	
PER1	79	Circadian clock	
PFAS	803	Purine biosynthesis	
PIEZO2	925	Ion channel	
PKHD1L1	191	Polycystic kidney	
PLEKHG5	653	Pleckstrin	
PLXNA1	1811	Plexin	
PLXNA2	1806	Plexin	
PLXNA3	1581	Plexin	
PLXND1	1815	Plexin	
PRUNE2	201		
PTPRG	870	Tyrosine phosphatase	
RAPGEF1	640	GTPases	
RAPH1	1044		
RBBP8	426	DNA endonuclease	
RREB1	1307	Zinc finger	
RUVBL1	422	ATPase	
SACS	517	Ataxia	
SCN9A	1332	Sodium channel	
SIPA1L3	1252	GTPase activation	
SLIT3	840	Cell migration	
SMG6	872	Telomere maintenance	
SMYD4	63	Lysine methyltransferase	
SUV420H1	864	Lysine methyltransferase	
TENM1	461	Teneurin	
TENM2	679	Teneurin	
TOP2A	798	DNA topoisomerase	
TTN	3957; 4331; 4613; 4898	Muscle contraction	
ZFHX3	434	Zinc finger	
ZNF541	19	Zinc finger	
ZNF594	162	Zinc finger	
