# Supplementary figures and images for: Lysine methyltransferase SETD6 modifies histones on a glycine-lysine motif
Source: Epigenetics. 2019 Aug 1;15(1-2):26–31. doi: 10.1080/15592294.2019.1649529 (PMC6961689; doi:10.1080/15592294.2019.1649529)

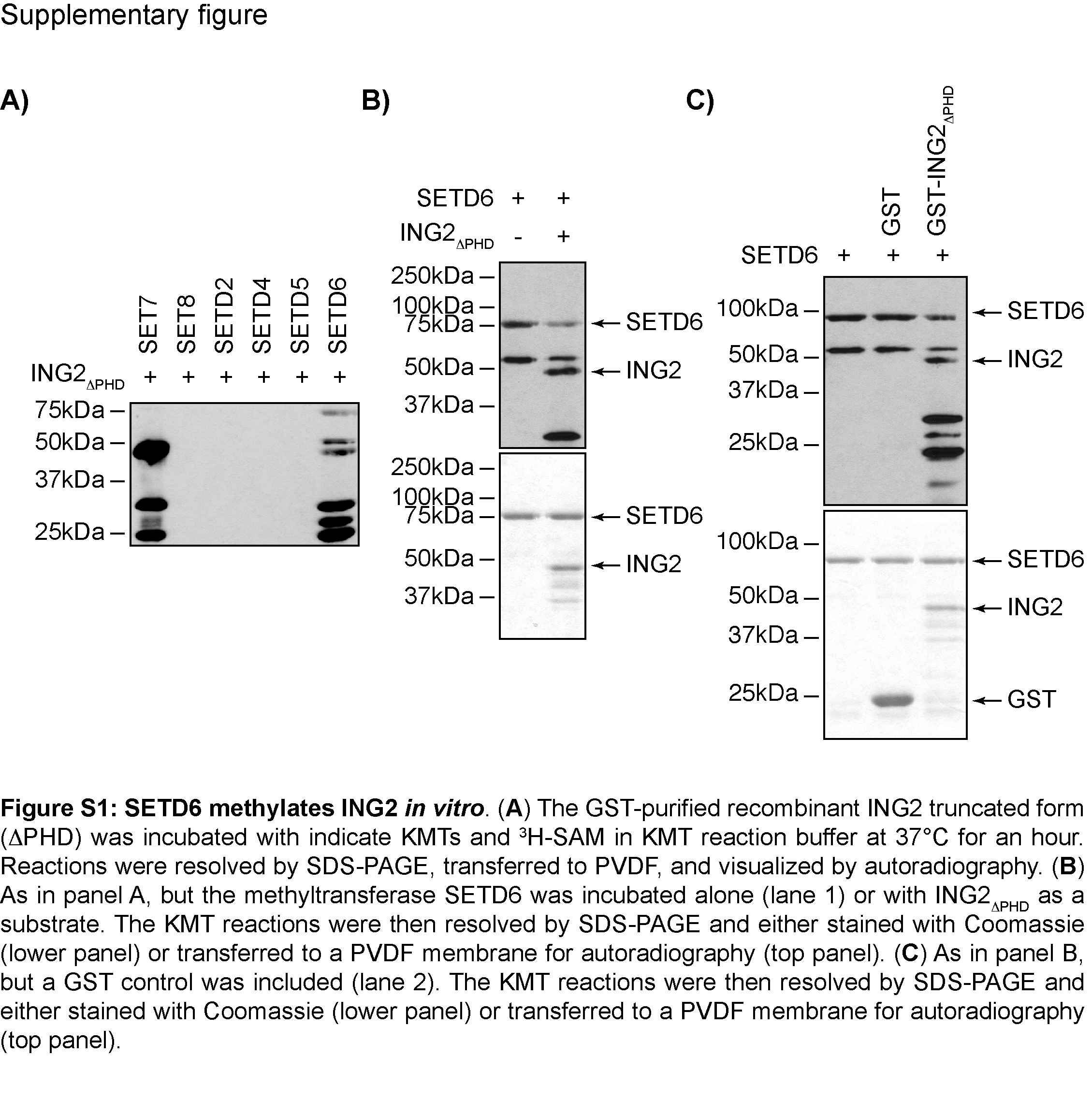

Supplement: Supplemental Material [file kepi-15-1-2-1649529-s002.tif]
